# Normothermic Machine Perfusion—Improving the Supply of Transplantable Livers for High-Risk Recipients

**DOI:** 10.3389/ti.2022.10460

**Published:** 2022-05-31

**Authors:** Angus Hann, Anisa Nutu, George Clarke, Ishaan Patel, Dimitri Sneiders, Ye H. Oo, Hermien Hartog, M. Thamara P. R. Perera

**Affiliations:** ^1^ The Liver Unit, Queen Elizabeth Hospital Birmingham, Birmingham, United Kingdom; ^2^ Centre for Liver and Gastrointestinal Research and NIHR Birmingham Biomedical Research Centre, Institute of Immunology and Immunotherapy, University of Birmingham, Birmingham, United Kingdom

**Keywords:** transplant, normothermic machine perfusion, liver, preservation, marginal, retransplant

## Abstract

The effectiveness of liver transplantation to cure numerous diseases, alleviate suffering, and improve patient survival has led to an ever increasing demand. Improvements in preoperative management, surgical technique, and postoperative care have allowed increasingly complicated and high-risk patients to be safely transplanted. As a result, many patients are safely transplanted in the modern era that would have been considered untransplantable in times gone by. Despite this, more gains are possible as the science behind transplantation is increasingly understood. Normothermic machine perfusion of liver grafts builds on these gains further by increasing the safe use of grafts with suboptimal features, through objective assessment of both hepatocyte and cholangiocyte function. This technology can minimize cold ischemia, but prolong total preservation time, with particular benefits for suboptimal grafts and surgically challenging recipients. In addition to more physiological and favorable preservation conditions for grafts with risk factors for poor outcome, the extended preservation time benefits operative logistics by allowing a careful explant and complicated vascular reconstruction when presented with challenging surgical scenarios. This technology represents a significant advancement in graft preservation techniques and the transplant community must continue to incorporate this technology to ensure the benefits of liver transplant are maximized.

## Introduction

Since the introduction of liver transplant as a treatment option for end stage liver disease and liver cancer in the 1960s, its role and potential benefits have expanded substantially (1, 2). In the initial phase of liver transplantation following the acceptance of the brain death concept (3), graft options were restricted to whole livers from deceased donors. In the modern day, this has expanded to include reduced size, auxiliary, domino, and split grafts from either deceased or living donors. The utilization of these different graft types has partly been driven by the fact that the accepted indications for liver transplant have also expanded over the last half century (4–6). Benefits from transplantation have been demonstrated in situations other than cirrhosis or primary liver cancer, including metabolic disease, colorectal liver metastases, and perihilar cholangiocarcinoma (6–8). Demand for organs continues to increase and exceeds the supply (9). Therefore, a proportion of patients on the waitlist miss their window of opportunity and are delisted. As an example, during 2018 in the United States (US), 18.6% (equating to 1471 patients) of the year’s starting waitlist was removed due to either death or becoming too sick to transplant (9). Maximizing graft utility is therefore paramount, and proven strategies, such as normothermic machine perfusion (NMP), should be routinely incorporated into clinical practice for certain graft-recipient scenarios.

**FIGURE 1:**
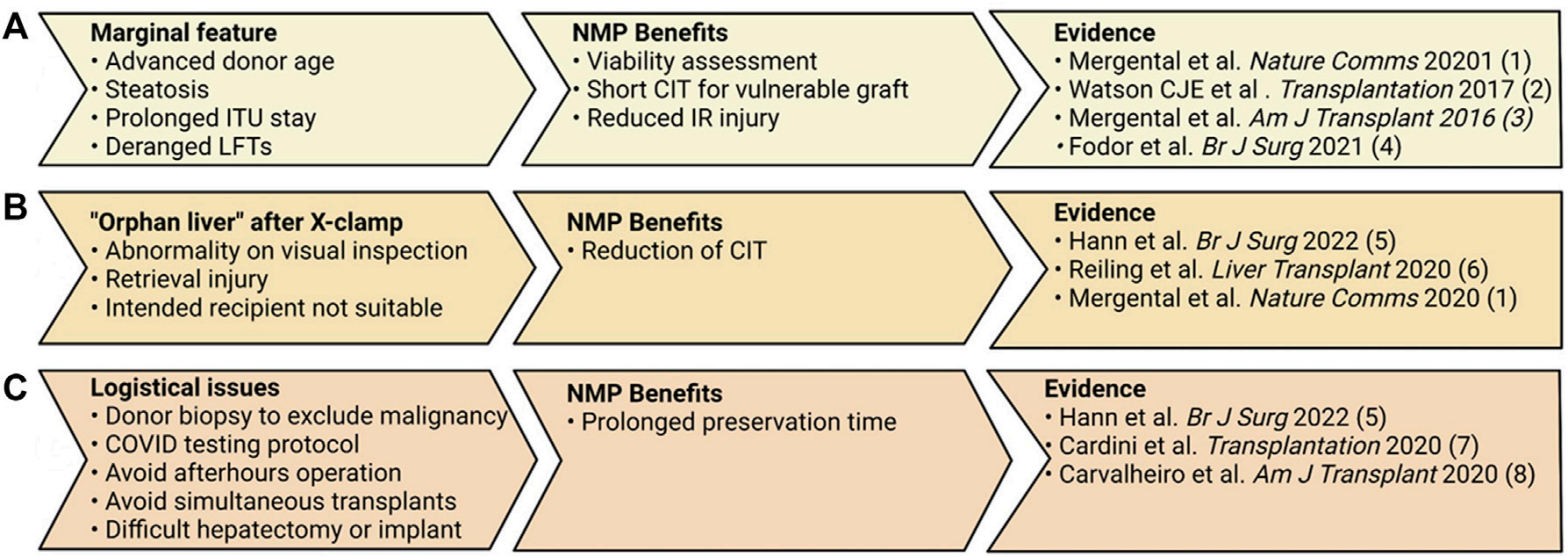
Three scenarios in which normothermic machine perfusion (NMP) can applied effectively by a transplant center. **(A)** In the setting of marginal graft features, NMP can shorten the CIT these grafts are subjected to and objectively assess the grafts’ function. **(B)** NMP is ideal for the situation in which an offer is received and CIT has already commenced, or one recipient is not deemed appropriate and more time is required to prepare another recipient. **(C)** Lengthening the preservation time can offer numerous benefits for the logistics around the transplant operation (12–14, 27, 30, 33, 40, 43).

The assessment that a patient, with an accepted indication, can be transplanted with a good outcome is reliant on social, psychological, medical, and surgical factors. Further to this, the risk - benefit assessment must be individualized as not all perspective recipients present with the same risks, nor should they all be expected to glean the same benefits. Despite research into overall risk (10, 11), the transplantability of each patient is largely subjective. Surgically high-risk recipients are those patients with certain factors that present additional obstacles, requiring the surgical team to adapt their strategy from that of a routine transplant. These may include extensive portal vein thrombosis, previous hepatobiliary surgery, or previous liver transplantation. In these situations, altered anatomy, obliterated tissue planes, and excessive bleeding may be encountered. There may also be the requirement for complex vascular reconstruction and flow modulation techniques for both the artery and portal vein. In combination, these factors increase the surgical insult, prolong the cold ischemic time (CIT) of a traditionally preserved liver graft waiting to be implanted, and may exacerbate the ischemia-reperfusion injury process. This may result in physiological instability and poor outcomes, especially if accompanied by early graft dysfunction. Historically, graft options for surgically high-risk patients were restricted to only those of optimal quality as they could withstand the longer cold ischemic period and provide immediate graft function. NMP can minimize CIT, objectively assess graft function, and safely prolong the preservation time (12, 13). We have recently published the results of our approach for retransplant candidates, which utilizes NMP for suboptimal grafts (14). In addition to facilitating the use of “orphan” grafts for a group that were disadvantaged by the current allocation scheme, it provides logistical benefits for the completion of a difficult operation (Figure 1) (14). The additional challenges imparted by the COVID-19 pandemic and the consequential recipient testing protocols and strain on intensive care resources has meant that our institution has been required to increasingly apply NMP for non-patient-related reasons. Although beneficial via other means, other machine perfusion techniques such as normothermic regional perfusion and hypothermic oxygenated machine perfusion do not offer the same advantage in operative logistics (15, 16).

Selecting donors and organs that can be safely used in the transplantation process is a challenging task. Organs chosen for transplantation should pose a minimal risk of donor-derived infection or malignancy, and ideally function in both the short and longer term. Graft-recipient matching goes beyond the size and ABO blood type compatibility, it relies on clinical judgement of the donor organ and its expected graft function, with the recipient stability to withstand the physiological disturbance caused by ischemia reperfusion injury (17). The majority of western countries, including the United Kingdom (UK), rely on deceased donors as the source of liver grafts and therefore operate with a limited supply of organs. This means difficult decisions need to be made regarding allocation of grafts (18). On the other hand, numerous deceased donor liver grafts are declined for transplantation each year, undoubtedly a portion of these would pose a significant risk to the recipient and truly are unsuitable (9). However, a proportion of these grafts may be suitable for transplantation with the use of machine preservation strategies such as NMP. In this article, we will further outline the reasons for using this preservation technology for high-risk recipients, and how NMP can assist in graft assessment.

## High Surgical Risk Recipients

A liver transplant is a challenging operation, with numerous risks due to both the technical aspects of the procedure and the recipients’ physiology. Despite advances in technique, such as preservation of the recipient inferior vena cava (2, 19) and reduction in perioperative blood loss, the surgical insult remains significant. Beyond the standard recipient with either acute liver failure or chronic end stage liver disease, additional technical factors may increase the challenge and risk for the surgeon and recipient, respectively. Previous hepatectomy or liver transplant, vascularized adhesions due to portal hypertension, and displacement of anatomical structures that all result in unclear dissection planes are such examples. These factors lengthen the hepatectomy phase and prolong the CIT of a graft if preserved *via* SCS. Endovascular stents and thrombi in major vascular structures or even extending into the right portion of the heart may require extensive logistics for use of a veno-venous bypass, and involvement of cardiothoracic teams. The ability of NMP to abbreviate the cold ischemic period but prolong the graft preservation in a safe manner provides a mechanism to expand graft options for these patients, accommodate the complex logistics, and mitigate the risk of unanticipated surgical factors.

It must be emphasized that with the combination of NMP, a high risk recipient, and a suboptimal graft, the implantation time must be kept short as the graft is already warm and therefore ATP depletion will proceed at a faster rate than following SCS. Scientific evidence for this is yet to be published, however more prolonged implantation times with NMP-preserved grafts are likely unfavorable. Alternative surgical strategies can be used in complex scenarios with NMP as opposed to cold storage. Rather than striving for a short cold ischemic period with the standard preservation method and early reperfusion of the graft, NMP allows time for a careful dissection to gain proper vascular control prior to the implantation phase and this is particularly useful in the setting of portal vein or late hepatic artery thrombosis when the implantation can be complicated (20). The length of this period has previously been shown to correlate with early graft function and long term outcome, with suboptimal grafts being impacted the most (21, 22). A portal cavernoma, fragile varices, and large spontaneous porto-systemic shunts may have developed following thrombosis of the portal vein. Retransplantation in the setting of late hepatic artery thrombosis is indicated when refractory or recurrent biliary sepsis occurs, often accompanied by inflamed tissues and infected bilomas. These obstacles must be overcome, and the implantation technique of the graft must ensure adequate inflow to the graft for a successful outcome (23). In the setting of late hepatic artery thrombosis, Buccholz et al. reported that aortic conduits were required in 83% of recipients (24). Regardless of whether this originates from a supraceliac or infrarenal position, the arterialization time reported in a multicenter study can be lengthy (23). The presence of a portal vein thrombus may necessitate extensive dissection in a caudad direction posterior to the pancreas to the splenic and superior mesenteric vein confluence, and a venous interposition graft if the native portal vein cannot be adequately thrombectomized. In an effort to keep the implantation and arterialization time to a minimum, we ensure the arterial and/or venous conduit is anastomosed on the proximal (recipient) side prior to disconnecting the graft from NMP and commencing the implantation.

Appropriate graft-recipient matching has been shown to be an important factor in ensuring optimal liver transplant outcomes, with marginal grafts generally preferred for recipients with lower model for end stage liver (MELD) scores (25). The rationale for this is that these recipients have a greater physiological reserve to tolerate an early period of graft dysfunction. Therefore, the suggestion that high-risk and complex recipients should be transplanted with marginal grafts contradicts the common belief that a premium quality liver graft will be required in these scenarios (26). This belief likely stems from many decades of experience with static cold storage in which the CIT was prolonged, resulting in more severe reperfusion injury and graft dysfunction as a consequence. The introduction of NMP has challenged this belief, with several successful reports of using suboptimal or “orphan” grafts for challenging cases (12, 14, 27–30). In a recent study from our center that compared a prospective group undergoing late liver retransplantation with suboptimal NMP-preserved grafts with two retrospective cold storage control groups, the outcomes were shown to be equivalent (14). Extrapolating the results from the high-risk group in this study, several of whom were undergoing a third graft, this approach can be employed for others that are complex from a surgical perspective. The utilization of NMP can therefore expand the proportion of grafts suitable for this high-risk group.

## Graft Assessment via Normothermic Machine Perfusion

**FIGURE 2:**
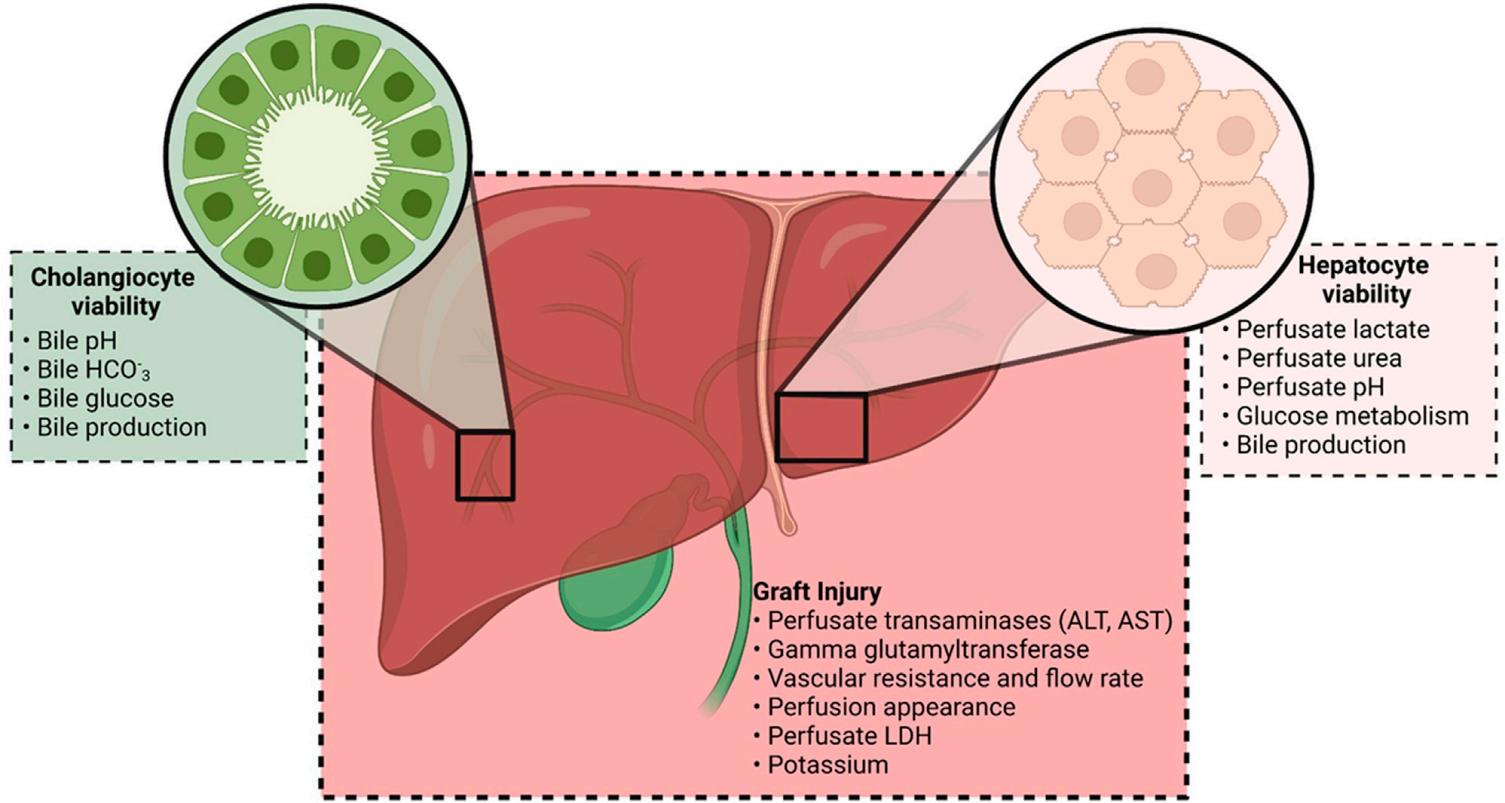
Markers currently being used by transplant centers to assess viability on the NMP circuit. Markers can be considered as indicating graft injury and cholangiocyte or hepatocyte viability. The combination of these markers is used in the viability criteria reported by different institutions.

Assessment of graft viability *via ex situ* machine perfusion remains an imperfect science, with many unanswered questions (31). Many different institutions have reported the criteria they apply to determine if a graft is viable, and these are being constantly altered to reflect the growing experience (12, 13, 28, 32–34). These have been extensively reviewed by other authors (35–37). The different markers reportedly used for graft assessment during NMP can be considered as surrogate indicators of graft injury and hepatocellular or cholangiocyte function (Figure 2). Graft injury sustained prior to NMP and poor hepatocellular function will manifest as severe graft dysfunction in the early post-operative period (38). Cholangiocyte injury sustained during the preservation process may be detectable through altered bile composition, and manifest as ischemic type biliary lesions (ITBLs). Although there is growing evidence that NMP can reduce the incidence of ITBLs, the risk of this complication is undoubtedly higher in DCD grafts (13, 39, 40).

Lactate clearance is the one viability marker that is consistent across all reported liver NMP viability criteria. Prior to connection of the liver to the NMP circuit which contains preserved human red blood cells, the lactate is usually high (10–20 mmol/L) due to the high concentrations of this substance within preserved bags of human erythrocytes (41). Following connection of a liver with functional hepatocytes, lactate is converted to pyruvate by lactate dehydrogenase in the hepatocyte cytosol (42). This lactate uptake and metabolism has been reported to occur predominantly in zone 1 hepatocytes which receive the best perfusion and oxygenation, therefore it has been proposed that a failure to clear lactate indicates an extensive and panlobular injury (35). Despite this, adequate lactate clearance and post operative graft function have been demonstrated in the setting of extensive hepatocyte necrosis (29). The normal pattern of lactate clearance on NMP is a rapid early phase, that slowly tapers off and reaches a plateau for the remaining period of perfusion (33, 43). This early change in lactate concentration is clinically useful as it gives an early indication of the likelihood the graft is transplantable as other indicators such as bile production and glucose metabolism generally become evident later. It has previously been demonstrated that some transplantable grafts do not follow the above “normal” pattern (28). Instead, they may demonstrate a slower but gradual decline or a slight increase in lactate concentration following the initial rapid decline (28). In these situations, we recommend continuing the duration of the perfusion to determine if the ability to clear lactate and maintain it at ≤2.5 mmol/L is possible. In our experience with using a closed circuit device (Organox®, Oxford, United Kingdom), we have noted that a rise in lactate (after previously rapid clearance) is often the result of bleeding from the liver hilum and this should be addressed. It may occur due to a larger proportion of blood returning directly to the reservoir bag, and therefore bypassing the metabolism and clearance by the hepatocyte. We apply the previously reported viability criteria regardless of the recipient, and also have reported successful outcomes recently with further expansion of the time cut-off for DBD grafts (13, 28).

## Expanding the Pool of Transplantable Grafts

Optimizing the safe utilization of deceased donor livers should be an ambition of all transplant programs. Despite recent improvements, the organ discard rate for retrieved organs from deceased donors in the US and UK is 8.4% and 18.2%, respectively (9, 44). Previous work has revealed concern regarding organ quality or donor history which accounts for the majority of transplant center declines (45, 46). Additional reasons for organ decline that emerge at the time of retrieval include the macroscopic appearance, a prolonged donor warm ischemic time in the setting of DCD donation, and unexpected laparotomy findings suggestive of malignancy. Despite sub-optimal donor features, many centers have reported acceptable results using livers “that nobody wants”, otherwise known as “orphan” livers (13, 27, 45, 46). This is a real-world demonstration that there is capacity to increase the “standard” acceptance criteria of liver grafts and NMP is a safe way to continue expanding these boundaries.

### Donor Medical History

The only absolute contraindications to transplanting a deceased donor’s liver based on their medical history is an established diagnosis of cirrhosis, primary central nervous system lymphoma, hematological or metastatic malignancy, active JC viral infection, or a transmissible spongiform encephalopathy (47, 48). The more common reasons in the current era for graft decline include donor age, alcohol history, abnormal liver function test results, and peri-mortem events (49, 50). Separating out the proportion of these grafts that can be safely transplanted is a challenging task, and we find NMP technology beneficial.

#### Donor Age

The physiological effects of age have less of an impact on the liver compared with the kidney and heart. However, both structural and functional differences exist in livers of older donors. The metabolic function of the hepatocyte has been shown to be decreased in the elderly. Peterson et al. demonstrated that the phosphorylation and oxidative capacity of mitochondria within the hepatocytes of the elderly (61–84 years) was reduced by 40% compared with young controls (18–39 years) (51). This was also accompanied by an increase in the triglyceride content of hepatocytes, which is further increased with an insulin-resistant state (51, 52). These deficiencies make the graft of an older donor less tolerant of the ischemic periods inherent in the transplant process. Structural changes that have been described in the livers of elderly people include increased fibrosis, possibly as the result of enhanced Th2 cytokine expression from macrophages (53).

The threshold for considering a donor to be of advanced age varies considerably, ranging from ≥40 to ≥80 years of age (54–56). Recent evidence demonstrates that elderly donors of DCD (>70 years) and DBD (>80 years) grafts can be used safely with 5 years graft survival rates of 74% and 77%, respectively, and therefore advanced age should not be a contraindication (54, 57). Grafts from older donors should be viewed as more susceptible to cold preservation injury for the aforementioned reason, therefore every effort should be made to minimize the CIT and NMP is an ideal strategy. Through the application of NMP on receipt of the graft at the recipient center, even recipients that will likely require a prolonged hepatectomy can be transplanted with a graft from an elderly donor with a short CIT.

#### Alcohol History

Alcohol-induced liver damage progresses in severity from hepatic steatosis to alcoholic steatohepatitis and then cirrhosis (58). Approximately 90% of individuals who consume excessive quantities of alcohol for at least 2 weeks, will develop macrovesicular hepatic steatosis (58). However, this will resolve following even a short period of abstinence but approximately one third of individuals with hepatic steatosis from alcohol will progress to steatohepatitis (58). Both the transaminases and gamma-glutamyl transferase (GGT) are relevant as they may give an indication of acute hepatocyte injury and steatosis, respectively (59).

The literature describing the transplant outcomes of donors with an excessive alcohol history is limited. Mangus et al. reported similar short and medium-term outcomes in groups of recipients that received a graft from either a donor with or without a history of excessive alcohol consumption (60). The graft recipients peak alanine aminotransferase (ALT) was higher in the excessive alcohol group, however the incidence of graft loss within the first 90 days was similar (6% vs. 7%, *p* = 0.75) (60). These authors analyzed the histological evidence provided from the post reperfusion biopsies of the excessive alcohol consumption group, and only 8% had fibrosis (any severity) and 9% had steatosis (>20%), respectively (60). However these authors defined excessive alcohol consumption as ≥2 more alcoholic drinks per day on a chronic basis (“at least several years”) (60). This may mean that these findings underestimate the risk in donors who may consume in excess of 10 alcoholic drinks per day, which is not uncommon in the United Kingdom. At our institution, donors with a history of excessive alcohol consumption are given full consideration if there is no established diagnosis of alcoholic liver disease. Visual inspection of the graft occurs at the time of retrieval and if concerns regarding significant steatosis or steatohepatitis exist, a graft biopsy is performed and our experienced liver histopathologists review the frozen sections before proceeding to transplantation. If the donor (and retrieval procedure) are occurring at a distant hospital, utilizing NMP provides additional time for histological and functional assessment.

#### Liver Function Tests

The commonly described “liver function tests”, actually provide minimal insight into the liver’s synthetic function, if at all. Despite this, even minimal elevations of liver enzymes are considered as criteria for defining marginal donors as per Eurotransplant criteria (61, 62). Alanine aminotransferase (ALT) is found predominantly in the cytoplasm of liver and kidney cells, whereas AST is found in both the cytoplasm and mitochondria of all cells (63). Therefore sources of elevation outside the liver, such as skeletal muscle damage and hemolysis, should be considered when considering a potential donor’s transaminases (63). In addition, factors such as hemodynamic instability, trauma, and sepsis may result in deranged liver function results through various mechanisms (50, 59, 64). Mangus et al. reported comparable outcomes in patients transplanted with grafts from donors with elevated peak ALT levels >1000 IU/L and 500–1000 IU/L when compared to those with peak ALT <500 IU/L. In the group with elevated peak ALT levels (≥500 IU/L), anoxia was the cause of death in a significantly higher proportion of patients compared to those with peak ALT <500 IU/L. Therefore, the authors concluded that an acute anoxic event likely accounted for the liver function derangement (50). Interestingly, the extent of necrosis on graft biopsy did not positively correlate with the peak ALT elevation (50). Adding to the argument that these type of donors are an acceptable way to expand the pool, data from the Scientific Registry of Transplant Recipients (US) demonstrate the discard rate being approximately 70% for a donor’s high transaminases (>1000 IU/L) as opposed to 22% for low transaminases (<1000 IU/L) (65).

The viability assessment provided by NMP is the ideal preservation platform for these grafts in the three aforementioned clinical scenarios that lead to organ discard, and has previously been shown to be effective even when the transaminase elevation is accompanied by significant hepatocyte necrosis (29). Our institution gives full consideration to the utilization of livers from donors with deranged transaminases, and places a greater importance on the trend rather than the peak level. The half life of AST and ALT in the human circulation differs, with it being approximately 17 (±5) h for AST and 47 (±10) h for ALT (63). Therefore following any transient insult to the liver during the terminal illness, it should be expected that the peak AST will be higher, occur earlier, and more rapidly decline than ALT, and therefore a decline in donor AST will be the first sign of a resolving insult. Donor bilirubin is undoubtedly an important biochemical test in the context of donor evaluation and is more useful in identifying donors that likely have a poorly functioning liver than transaminases. In support of this belief, it was the only biochemical variable identified as having a significant relationship to transplant outcome in the construction of a UK donor liver quality model by Collet et al. (66). In the absence of an extrahepatic cause of donor hyperbilirubinemia, our upper limit of acceptance for donor bilirubin is 2 x the upper limit of normal (40 μmol/L, 2.4 mg/dl). In the series of 14 donors with transaminases >1000 IU/L reported by Martins et al., only one had a peak bilirubin above this value. Other causes of jaundice in the donor such as hemolysis or Gilbert syndrome (UDP glucoronosyltransferase-1A1 deficiency) should be considered, and useful additional tests such as lactate dehydrogenase and conjugated and unconjugated bilirubin should be requested.

#### Peri-Mortem Events

**FIGURE 3:**
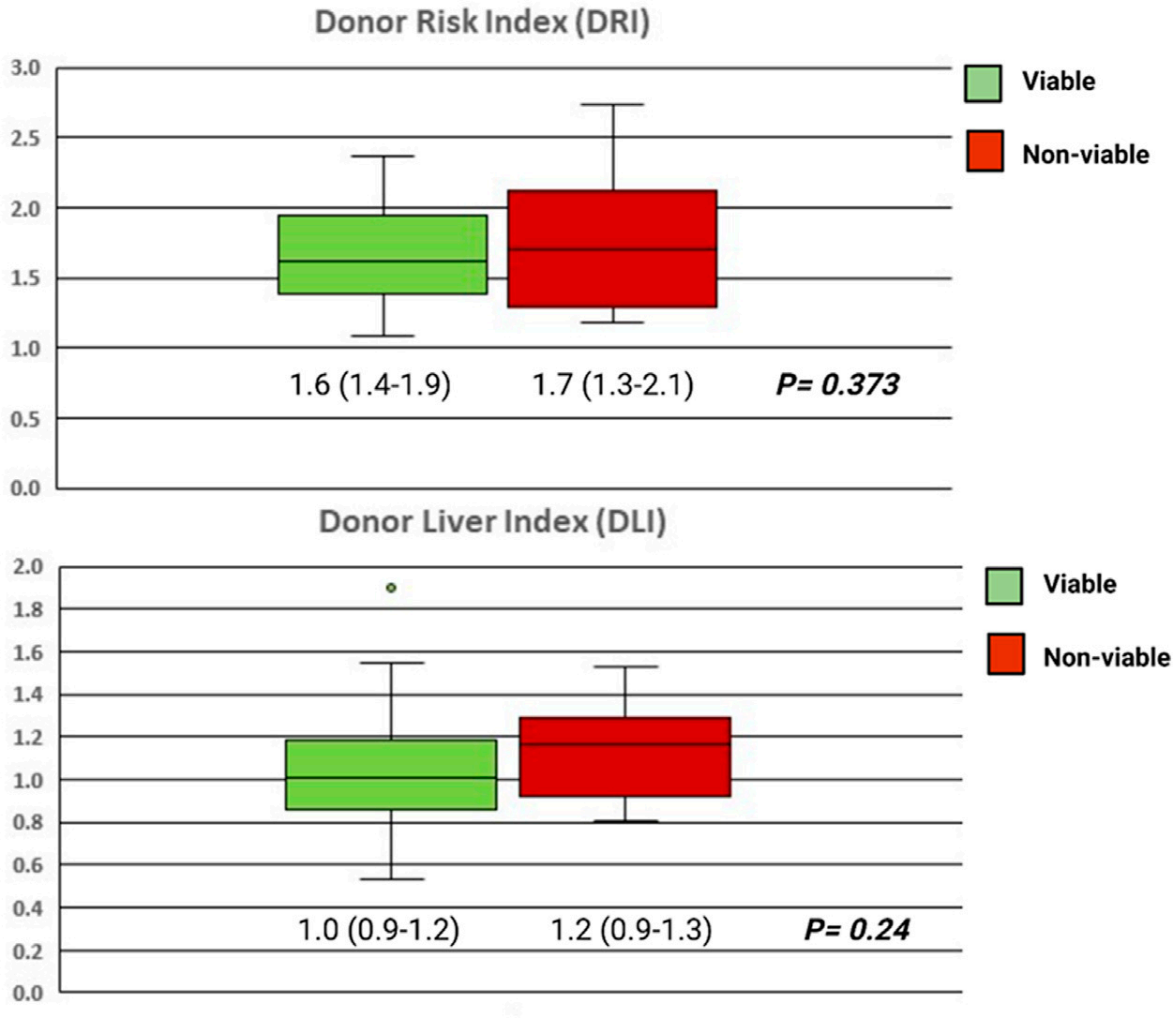
Box plots showing the DRI and DLI of the grafts that were assessed as viable and transplanted following NMP (green, *n* = 95), and those that were assessed as non-viable (red, *n* = 12). Groups compared with the Mann-Whitney U test and independent samples T-test for the DRI and DLI, respectively.

In the context of deceased organ donation, consideration of the events and pathological process that resulted in brain death (or the consideration for DCD donation) is required. Consensus guidelines are available for the scenario of donor malignancy or infection and are beyond the scope of this review (47, 48). In a seminal publication by Feng et al. using US registry data, which reported the donor risk index (DRI), death from trauma or hypoxic brain injury (HBI) was associated with better outcomes than cerebrovascular accident or other causes (55). However, this finding was not replicated with UK data in derivation of the donor liver index (DLI) (66). Despite these statistical models taking into account several donor and graft features, no significant difference exists between those that satisfied our institution’s viability criteria and those that did not (Figure 3).

A donor ICU length of stay >7 days is considered an extended criteria donor by Eurotransplant, whereas previous consensus meetings have stipulated an ICU length of stay <5 days is ideal for DCD donors (59, 62). It seems that a consensus on what comprises a prolonged donor ICU stay is yet to be reached (67). A longer ICU stay does appear to be associated with increased donor infections, however a positive culture in the donor does not appear to influence the recipient’s outcome (67, 68). In a study by Misar et al., which compared the outcomes of pediatric recipients who received a graft from a donor following a short (<5 days) or long (≥5 days) ICU stay, there was no difference in overall graft or patient survival (67). In our practice, we agree with the notion put forward by Strasberg et al. in which it is not the length of ICU stay per se that is deleterious, rather the events that transpire during this period (69). These could include hypotensive episodes, sepsis, exposure to hepatotoxic medications, surgical procedures, and lack of adequate nutritional support.

A blunt traumatic injury may result in serious intracranial injuries and brain death as a result. There may also be an associated traumatic liver injury, which is most commonly lacerations or contusions of the liver parenchyma (70). These traumatically injured livers may still be transplantable and certain strategies, including a back table graft reduction, have been reported with success (70). A concern with utilizing NMP in this scenario is that uncontrollable bleeding may occur on the perfusion circuit due to the high concentration of heparin and lack of coagulation factors. In general, we have seldom had problems with bleeding on the NMP machine from traumatic injuries sustained prior to or at the time of organ retrieval. Application of gauze swabs and hemostatic products have been used to control minor bleeding from capsular damage.

### Graft Steatosis

A frequent reason for a graft to be assessed as suboptimal and discarded is the presence of steatosis (71). Although the cause of hepatic steatosis is likely multifactorial, there is a strong association with body mass index (BMI) (72). This presents a significant issue for the field of liver transplantation as the western population is becoming more obese. Therefore, transplant clinicians must aim to better understand the implications and limitations of steatotic livers and further develop effective medical and surgical strategies to facilitate their safe utilization.

Multiple studies have demonstrated that even severe microsteatosis does not have a deleterious effect on transplant outcome (73–75). It is the extent of macrovesicular steatosis which is the main concern of the transplant surgeon, as an association with primary non-function and reduced graft survival has been demonstrated in the past (76, 77). Despite graft biopsy and fresh frozen histological assessment being considered the gold standard (71), the method (visual inspection or histology) used to assess graft steatosis is variable. As an example, the UK organ donation service does not have pathology services available after-hours and therefore retrieval surgeons rely on visual inspection and palpation. Furthermore, the implanting surgeon is limited to photographic assessment alone. A limitation of visual assessment and palpation of graft texture is the inability to reliably distinguish microvesicular from macrovesicular change. In a publication by Yersiz et al. that compared surgeons’ visual assessment to pathologists’ microscopic assessment, the positive predictive value of a surgeons’ assessment of >30% macrovesicular steatosis as per histology was only 52% (78). It must also be noted that histological assessment also has its own limitations including interpretation differences between pathologists (79).

The extent of macrosteatosis that represents a graft that is totally unusable remains unclear (80). Studies have frequently failed to define the type of steatosis, making it difficult to draw conclusions draw given the aforementioned differing implications (79, 80). Nevertheless, it is well known by liver transplant clinicians that recipients of livers with moderate to severe steatosis have a more turbulent early post-operative period. In general, more than 30% of macrosteatosis is viewed as increasing the risk of clinically relevant early graft dysfunction. Safely utilizing steatotic livers will become increasingly important due to the obesity epidemic. As described by Jackson et al., drawing comparisons between outcomes of steatotic and non-steatotic livers is most often meaningless as the patient (and surgeon) do not get to decide simultaneous offers of each (81). Therefore the risk-benefit decision is really between survival with a steatotic transplanted liver, and no transplant at all. In a large US study that used national registry data, waitlisted recipients for whom a steatotic liver (≥30% macrosteatosis) offer was made but declined, 22.8% died on the waitlist and 17.6% were delisted. Overall, accepting a steatotic donor liver offer reduced the risk of mortality by 62% in comparison to declining it (81). Furthermore, the reduction in mortality seen with receiving a steatotic liver was greatest in the subgroup with the highest MELD score (81). Further demonstrating that steatotic livers offer a survival benefit in even the sickest of patients.

NMP has previously demonstrated benefits in the setting of steatotic livers, and probably represents one of the most frequent indications for its use (12, 13, 82). The experience with NMP preservation and transplantation of steatotic livers appears to be accruing, and the viability assessment provided by this modality clearly has a role in distinguishing those that will function adequately in the early post operative period. Our center’s experience is that one of the main reasons for a graft to be “orphaned” is steatosis, and only severely (>60% macrovesicular) steatotic livers fail the viability assessment. Minimizing the CIT of these grafts is paramount for a good outcome and NMP can facilitate this.

### Prolonged Cold Ischemic Time

Reducing the cellular temperature to approximately 4°C slows cellular metabolism and therefore slows ATP consumption to approximately 10% of activity at normal body temperature (83). Nevertheless, ATP consumption continues and advanced donor age, BMI, and poor nutritional status may result in lower baseline ATP levels and therefore the organs tolerate the ischemic period poorly. The hypothermic conditions result in direct injury to cellular structures, such as the cytoskeleton and various organelles (84). Therefore the energy-conserving effect of cold preservation is partly offset by it non-physiological nature. An ischemia-reperfusion injury is the consequence of this preservation period and this impacts early graft function, and is the main reason for primary non-function (38). An improved understanding of the effect a cold ischemic period has on outcomes may have lead to the shortening of the cold ischemic period that is evident in registry data, and represents one of the main drivers for machine preservation technology (9).

Feng et al. demonstrated that in reference to a graft with an 8 h CIT, every additional hour resulted in a 1% decrease in 1 year graft survival (55). This suggests that the beneficial effect of minimizing CIT demonstrates a continuous pattern, rather than one with a clear threshold effect. Although NMP applied in a back-to-base model cannot alter the CIT associated with the donor procedure, it can abrogate the CIT that is associated with a lengthy recipient preparation period or hepatectomy. In our institution, we aim to keep the CIT at less than 6 h when applying NMP to suboptimal livers. This is achievable in the majority of instances, even if the graft is accepted very late in the retrieval process, as long as adequate personnel are available to prepare both the machine and the graft for perfusion. The timespan from arrival of the graft at our center until the commencement of NMP is approximately 2 h and this should be considered in the estimates of CIT.

## Normothermic Machine Perfusion for High-Risk Graft-Recipient Combinations

**FIGURE 4:**
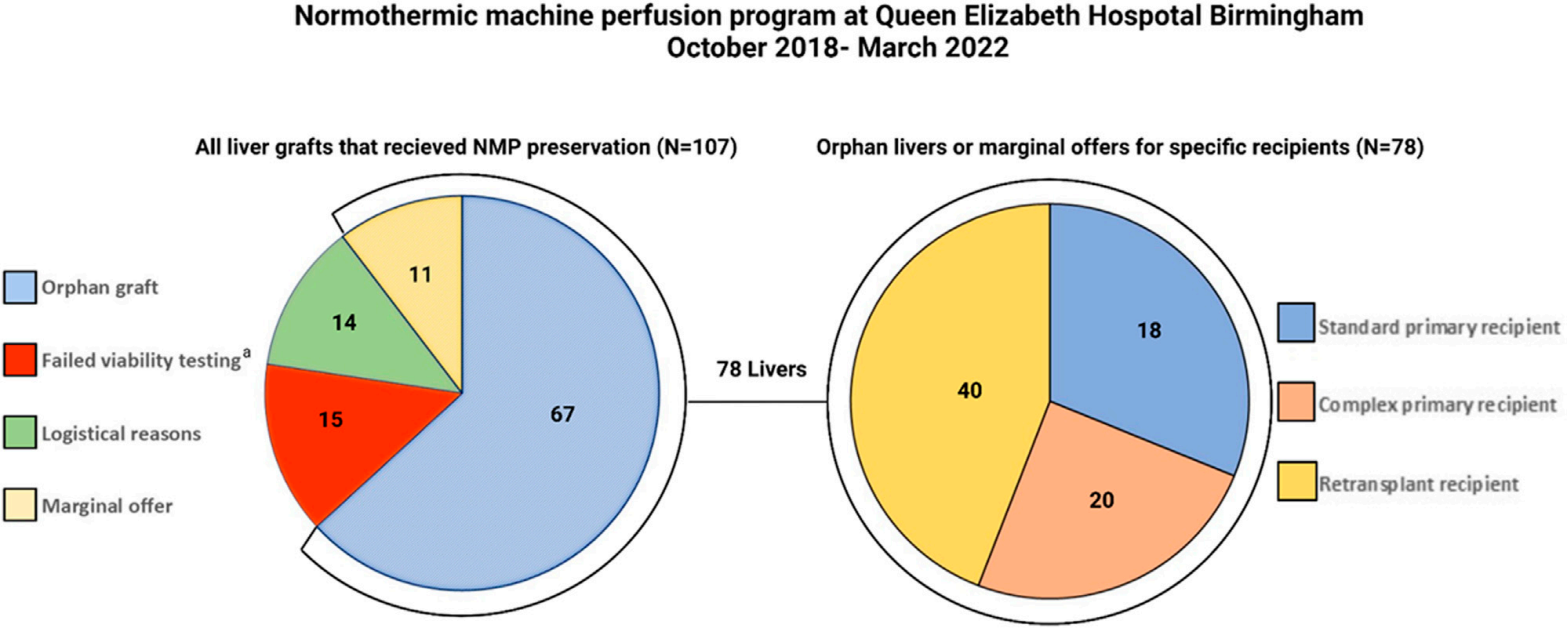
Pie charts demonstrating all grafts that underwent normothermic machine perfusion (NMP) at QEHB between October 2018 and April 2022 (left). The high-risk recipient-specific offers with marginal features (*n* = 11) or “orphan” grafts (*n* = 67) comprised 78/92 (85%) of the grafts transplanted. The right pie chart demonstrates that the majority of the livers went to either retransplant (40/78, 51%) or complex primary transplant recipients (18/78, 23%). Complex primary transplant recipients were those recipients with previous major hepatobiliary surgery or Yerdel grade ≥III portal vein thrombosis. ^a^Two grafts not transplanted due to recipient reasons and one due to equipment failure.

In this section we discuss how we tie up the previously discussed issues together—the novel technology of NMP and the utility of this in the context of high-risk recipients who are significantly disadvantaged due to the scarcity of good-quality organs and timely transplantation of these candidates with marginal grafts. At our institution, Queen Elizabeth Hospital Birmingham, we have incorporated NMP technology into our service on a selected basis for the high-risk graft-recipient combination. The overall benefit of NMP technology over and above cold storage for standard graft-recipient combinations remains debatable. Although NMP is known to mitigate ischemia reperfusion injury and somewhat mitigate short-term beneficial outcomes, it is our belief that NMP should be utilized with a greater aim. Therefore, we utilize NMP in a manner that will allow high-risk recipients to benefit through improved graft access. The results of this approach have been previously published and presented at international conferences (14, 20). To update the current status of our program, between October 2018 and March 2022, we have perfused 107 liver grafts and 92 of these have proceeded to transplantation (Figure 4). Delivering a much needed organ transplantation service during the COVID-19 pandemic has provided additional challenges, however NMP has proven useful to overcome COVID testing requirements and hospital logistics regarding bed access. NMP provided a prolonged preservation time that would not have been possible, even in the standard graft-recipient combinations (85).

The ability to accept and safely transplant marginal and ‘orphan’ livers into high-risk recipients has been made possible with NMP due to a number of reasons. Firstly, the NMP viability assessment of a graft provides additional confidence that the graft will function adequately in the early post operative period. This is supported by the fact that PNF has not occurred in any of the 92 recipients of a liver preserved with NMP. Secondly, the ability to inspect and connect the liver to NMP expediently on arrival at our center allows the CIT to be kept to an acceptable minimum. This means that grafts that are declined at other transplant centers, in other parts of the UK, can still be reperfused within an adequate time period. The additional insult of a prolonged CIT on a graft with marginal attributes should not be underestimated, as undoubtedly there is an interaction between these variables as described previously. Finally, with the current national organ retrieval system in the UK being relatively inflexible about donor retrieval timing, it allows for a difficult operation to be performed during daylight hours with adequate and appropriate staffing.

**FIGURE 5:**
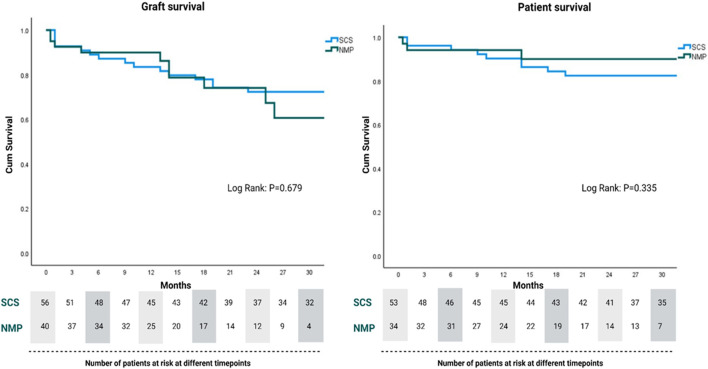
Graft and patient survival for normothermic machine perfusion (NMP) and static cold storage (SCS)-preserved grafts for retransplant recipients.

In regards to graft physiology following NMP, we have experienced a lower than expected incidence of reperfusion syndrome and clinically significant early allograft dysfunction than would be expected given the graft and recipient characteristics (86). In the cohort of NMP-preserved grafts depicted in Figure 4, 40 out of the 78 marginal grafts were transplanted into patients undergoing retransplantation. The outcomes of the initial 26 patients in this cohort have been published previously (14). Since completing enrolment in this previous study and the reassuring results, we have performed an additional 14 retransplants using NMP-preserved grafts and all of these have reached at least 3 months follow-up. Therefore we have experience with a unique cohort of 40 patients undergoing retransplantation with NMP-preserved marginal liver grafts. The donor and graft characteristics of this group, in comparison to a retrospective cold storage control cohort transplanted at our institution over the last 5 years, are demonstrated in Table 1. Similar to our previously reported findings, the graft and patient survival did not differ between groups (Figure 5) despite the NMP group having significantly more steatotic grafts (moderate steatosis; 30% vs. 4%, P=<0.01), donors with peak ALT >1000 IU/L, and grafts declined by at least one other transplant center (78% vs. 26%, *P=*<0.01). Furthermore, in this expanded cohort, the peak alanine transaminase in the initial post operative 7 days was significantly lower (521 IU vs. 796 IU, *p* = 0.02) in the NMP group, and the EAD rate was not significantly different (47% vs. 37%). The rate of early acute rejection was higher (50% vs. 27%, *p* = 0.02) in the NMP cohort and has been discussed in a previous publication. We acknowledge that the incidence EAD rate is higher than previous reports, however this did not translate to early graft loss. Furthermore, a large majority were “biochemical” EAD due to raised bilirubin only (>177 mmol/L) in the presence of severe immune-mediated rejection, without any clinically relevant organ failure.

**TABLE 1:** Donor, graft and recipient characteristics.

Donor	SCS group (N = 56)	NMP group (N = 40)	*P*
Donor age, (IQR)	52 (44–69)	50 (42–56)	0.67
Female	25 (45%)	27 (67%)	0.03
Donor BMI (IQR)	24.9 (22.5–28.3)	24.1 (21.4–27.7)	
Days in ICU (IQR)	2 (2–4)	3 (2–5)	0.22
DRI (IQR)	1.55 (1.40–1.73)	1.57 (1.38–1.69)	0.92
DLI (IQR)	1.05 (0.92–1.21)	0.99 (0.86–1.13)	0.16
Inotrope requirement	48 (86%)	36 (90%)	0.53
Smoker			
History of alcohol excess	11 (20%)	15 (38%)	0.07
Donor cardiac arrest	24 (44%)	13 (32%)	0.27
Downtime minutes (IQR)	30 (8–48)	38 (28–52)	0.142
Liver biochemistry			
Peak ALT, IU/L (IQR)	53 (21–99)	109 (40–669)	<0.01
Peak bilirubin, mg/dL (IQR)	9 (7–16)	13 (8–20)	0.03
Donor ALT ≥1000 IU/L	0 (0%)	9 (23%)	<0.01
**Graft**	**SCS group (N = 56)**	**NMP group (N = 40)**	***P***
Declined by at least 1 other center^a^	14 (26%)	31 (78%)	<0.01
Steatosis			<0.01
None	40 (73%)	21 (53%)	
Mild	13 (24%)	7 (18%)	
Moderate	2 (4%)	12 (30%)	
Cold ischemic time, min (IQR)	482 (409–596)	372 (325–425)	<0.01
Perfusion time, min (IQR)	—	759 (488–953)	N/A
Total preservation^b^, min (IQR)	482 (409–596)	1107 (746–1330)	<0.01
**Recipient**	**SCS group (N = 56)**	**NMP group (N = 40)**	***P***
Age (IQR)	43 (29–56)	36 (24–50)	0.05
UKELD	58 (55–63)	58 (53–61)	*0.73*
MELD	19 (14–25)	21 (13–26)	*0.82*
Number of previous grafts			0.06
One (first retransplant)	49 (87%)	29 (72%)	
Two (second retransplant	7 (13%)	9 (21%)	
Three (third retransplant)	0 (0%)	2 (7%)	
Indication			*0.40*
Hepatic artery thrombosis	17 (30%)	14 (35%)	
Chronic rejection	5 (9%)	8 (20%)	
Biliary complications	18 (32%)	9 (22%)	
Disease recurrence	13 (23%)	6 (15%)	
Waitlist duration (days)	72 (26–151)	235 (60–423)	*<0.01*
Follow up (Median, months)	40 (25–56)	21 (11–29)	<0.01

Categorical variables compared with Chi-square test. Independent sample T-test used to compare continues variables that were normally distributed. Mann -Whitney U test used to compare normally distributed continuous variables. AL, alanine aminotransferase; BMI, body mass index; ICU, intensive care unit; DRI, donor risk index; DLI, donor liver index; UKELD, United Kingdom model for end stage liver disease; MELD, Model for end stage liver disease; SCS, Static cold storage; NMP, Normothermic machine Perfusion.

a Reason related to donor or graft quality.

b Total preservation time comprised cold ischemic time and perfusion time.

## Conclusion

The transplantability of a liver graft has long been a subjective assessment. It is understandable that surgeons may therefore err on the side of caution, in an effort to do no harm. This is of course particularly important when the predicted surgical risks are already high. The ability of NMP technology to extend liver graft preservation time can allow more complex recipients to be transplanted in a controlled and safe manner. Furthermore, it can safely facilitate the usage of liver grafts for these recipients that would otherwise not have been considered. Evidence supporting the use of NMP for marginal organs and high-risk recipients is starting to emerge with promising data in the retransplantation setting. The problem inherent with researching this topic is the lack of appropriate controls for comparison. Retrospective control cohorts, or even propensity matching, have their limitations as under different preservation conditions these grafts would not have been transplanted previously (87). The benefits of NMP in the setting of a suboptimal graft and high-risk recipient justify the additional resources required for this technology. Similar to the improved understanding of cold preservation techniques throughout the second half of the 20th century, the application of NMP continues to be refined through research at many centers around the world. As experience with machine perfusion technology grows, this will hopefully translate into improved transplant outcomes.

## Data Sharing Agreement

Data collected for this study, including individual participant data and the data dictionary, will be made available to others at publication. The data will be in an anonymized form to protect participants’ privacy. The authorship agrees to provide access to all additional study documents.

## Abbreviations

ALT, alanine aminotransferase; AST, aspartate aminotransferase; BMI, body mass index; CIT, cold ischemic time; DBD, deceased brain death; DCD, deceased circulatory death; DLI, donor liver index; DRI, donor risk index; GGT, gamma-glutamyl transferase; HBI, hypoxic brain injury; HCV, hepatitis C virus; ICU, intensive care unit; ITBL, ischemic-type biliary lesions; MELD, model of end stage liver disease; NMP, normothermic machine perfusion; PNF, primary non-function; SCS, static cold storage; UK, United Kingdom; UKELD, United Kingdom model of end stage liver disease; US, United States.
